# Prior pneumococcal vaccination improves in-hospital mortality among elderly population hospitalized due to community-acquired pneumonia

**DOI:** 10.1186/s12890-024-02928-8

**Published:** 2024-04-08

**Authors:** Seohyun Kim, Moon Jin Kim, Jun-Pyo Myong, Yun-Hee Lee, Bo Yeon Kim, Ahyoung Hwang, Gui Ok Kim, Sung Hwan Jeong, Hyoung Kyu Yoon, Tai Joon An, Jeong Uk Lim

**Affiliations:** 1 Division of Pulmonary and Critical Care Medicine, Department of Internal medicine, Yeouido St. Mary`s Hospital, College of Medicine, The Catholic University of Korea, Seoul, Korea; 2 Department of Occupational and Environmental Medicine, Seoul St. Mary`s Hospital, College of Medicine, The Catholic University of Korea, Seoul, Korea; 3 Department of Urology, Seoul St. Mary`s Hospital, College of Medicine, The Catholic University of Korea, Seoul, Korea; 4 Healthcare Review and Assessment Committee, Health Insurance Review and Assessment Service , Wonju, Korea; 5 Quality Assessment Department, Health Insurance Review and Assessment Service , Wonju, Korea; 6 Quality Assessment Administration Department, Health Insurance Review and Assessment Service , Wonju, Korea; 7 Division of Pulmonary, Allergy and Critical Care Medicine, Department of Internal Medicine, Gil Medical Center, Gachon University, Incheon, Korea

**Keywords:** Community-acquired pneumonia, Pneumococcus, Vaccine, Mortality

## Abstract

**Background:**

Pneumococcal vaccination is a preventive method to reduce pneumonia related mortality. However, real-world data on efficacy of the pneumococcal vaccine in reducing mortality is lacking, especially in elderly patients. This study was conducted to assess the effects of prior pneumococcal vaccination in elderly pneumonia patients.

**Methods:**

The data was procured from the Health Insurance Review and Assessment and Quality Assessment database. Hospitalized patients who met the criteria of community-acquired pneumonia (CAP) were included and they were grouped according to vaccination state. Patients were aged ≥ 65 years and treated with beta-lactam, quinolone, or macrolide. Patients were excluded when treatment outcomes were unknown.

**Results:**

A total of 4515 patients were evaluated, and 1609 (35.6%) of them were vaccinated prior to hospitalization. Mean age was 77.0 [71.0;82.0], 54.2% of them were male, and mean Charlson comorbidity index (CCI) was 3.0. The patients in the vaccinated group were younger than those in the unvaccinated group (76.0 vs. 78.0 years; *P* < 0.001), and showed higher in-hospital improvement (97.6 vs. 95.0%; *P* < 0.001) and lower 30-day mortality (2.6 vs. 5.3%; *P* < 0.001). After adjusting confounding factors such as age, gender, CURB score and CCI score, the vaccinated group demonstrated a significant reduction in 30-day mortality (hazard ratio [HR] 0.58, 95% confidence interval [CI] 0.41–0.81; *P* < 0.01) and in-hospital mortality (HR 0.53, 95% CI0.37–0.78; *P* < 0.001) compared to the unvaccinated group in multivariate analysis. Vaccinated group showed better 30-day survival than those in non-vaccinated group (log-rank test < 0.05).

**Conclusions:**

Among elderly hospitalized CAP patients, prior pneumococcal vaccination was associated with improved in-hospital mortality and 30-day mortality.

## Introduction

Pneumonia is a one of the most common causes of hospitalization among the elderly and can be fatal, with in-hospital mortality ranging from 6 to 18% [1, 2]. Pneumococcal bacterium is a major pathogen which may lead to hospitalization through community-acquired pneumonia (CAP) [3–5]. Pneumococcal vaccine has been used as a preventive method to reduce pneumonia-related mortality, especially in elderly populations. A randomized, double-blind, placebo-controlled trial including 84,496 adults showed that 13-valent polysaccharide conjugate vaccine (PCV13) was effective in preventing vaccine-type pneumococcal, bacteremic, and nonbacteremic CAP and vaccine-type invasive pneumococcal disease [6, 7].

In South Korea, healthy adults 65 years or older are supported towards vaccination with 23-valent pneumococcal polysaccharide vaccine (PPV23) as a policy, and recommended for PCV13 as desired [8]. A national immunization program providing PPV23 to older adults aged ≥ 65 years was initiated in 2013 in South Korea [9]. However, real life data of efficacy of pneumococcal vaccine in reducing in-hospital mortality is lacking. There are some multicenter studies evaluating impact of pneumococcal vaccination in invasive pneumococcal diseases (IPD) [10, 11]. However, few nationwide or large-sized studies have been performed in South Korea.

In South Korea, quality assessment (QA) research is performed regularly to analyze appropriateness of CAP treatment. The QA program comprises the scores from eight categories, with pneumococcal vaccination history included as one of these categories. Utilizing the large-sized CAP database acquired from the nationwide program, this study was performed to assess efficacy of prior pneumococcal vaccine in reducing in-hospital mortality, 30-day mortality, and hospital stay in the elderly population admitted due to pneumonia.

## Materials and methods

### Data sources

This study used nationwide cross-sectional data for admitted CAP patients. After a procurement of official assignment of Joint Project on Quality Assessment Research from the Health Insurance Review and Assessment Service (HIRA), access to the QA database was granted for the purpose of analyzing the factors associated with the prognosis of CAP in South Korea. The QA database was collected and managed by the HIRA, and included all hospitalized patients with CAP in South Korea. The QA data tables contain basic demographic information of the patients with CAP and several core measure scores for CAP [12, 13]. All medical claims data were analyzed by the HIRA [14].

### Study population

The QA database analyzed in the study included the data reported from the 3rd QA (October 2017 ?∼ December 2017). The patients who met the criteria of being hospitalized due to CAP were included. Inclusion and exclusion criteria were as follows [12]:

#### Inclusion criteria

1. Patients aged ≥ 65 years
2. Patients who had at least one diagnosis code of pneumonia (J11.x ?∼ J18.x as primary or diagnosis within 2nd position) from the International Classification of Disease–Tenth Revision (ICD 10th )
3. Patients who were treated using at least 3 days of intravenous (IV) antibiotics

#### Exclusion criteria

1. Patients who had the following aspects not meeting the criteria of CAP: (a) hospital acquired pneumonia, (b) ventilator-associated pneumonia, (c) pneumonia developing during postoperative period, (d) patients who had been transferred previously from other health care facilities, and (e) patients who were hospitalized for more than 2 days in the 90 days prior to their admission by CAP.
2. Patients who were not treated with IV antibiotics first at the hospitals, such as those who were transferred from other medical facilities with prior antibiotics use or who were not administered antibiotics within 72 hours of admission.
3. Patients who underwent delayed pneumonia treatment due to inevitable causes such as emergent operation.
4. Patients who had the following conditions: (a) recently diagnosed with malignant cancer (≤ 3 months), (b) received chemo- or radiation therapy within the previous 3 months, (c) taking immunosuppressant agents, and (d) were previously treated with high dose steroids with a composition greater than 20 mg/day (≥ 14 days).

### Comorbidities

The Charlson Comorbidity Index (CCI) was calculated following the previous related articles [15, 16]. Classification of each comorbidity was based on the ICD 10th diagnosis codes for predicting the prognosis [17]. The HIRA database was matched with the QA database for records from one year prior to index date of each pneumonia case, using the lists of diagnoses to ensure accuracy. Information on smoking history was obtained from the interview records taken at the time of admission for CAP.

### Pneumococcal vaccination

The pneumococcal vaccination status was ascertained through a review of the patients’ medical histories. Recording vaccination history was a basic category of the QA for determining the appropriateness of CAP treatment in healthcare facilities. Patients with CAP were queried about their vaccination status at the time of admission. However, it was not possible to distinguish between those vaccinated with PCV13 or PPV23. Based on the information gathered during admission interviews, patients were categorized into two groups: unvaccinated and ever-vaccinated (either with PCV13 or PPV23).

### Statistical analysis

We used the analysis of student T-test and chi-square test for independence to compare the differences in the continuous and categorical variables between the two groups. Simple and multiple linear regression analyses were used to find the factors significantly affecting the hospital length of stay (LOS). Univariable and multivariable logistic regression analyses were performed to evaluate the factors associated with in-hospital and 30-day mortality. A *P*-value of < 0.05 was considered statistically significant. All the statistical analyses were performed using *RStudio Team (2020) (RStudio: Integrated Development for R. RStudio, PBC, Boston, MA URL*http://www.rstudio.com/.*)*

## Results

### Comparison of clinical characteristics between pneumococcal vaccinated and unvaccinated elderly patients

**Fig. 1:**
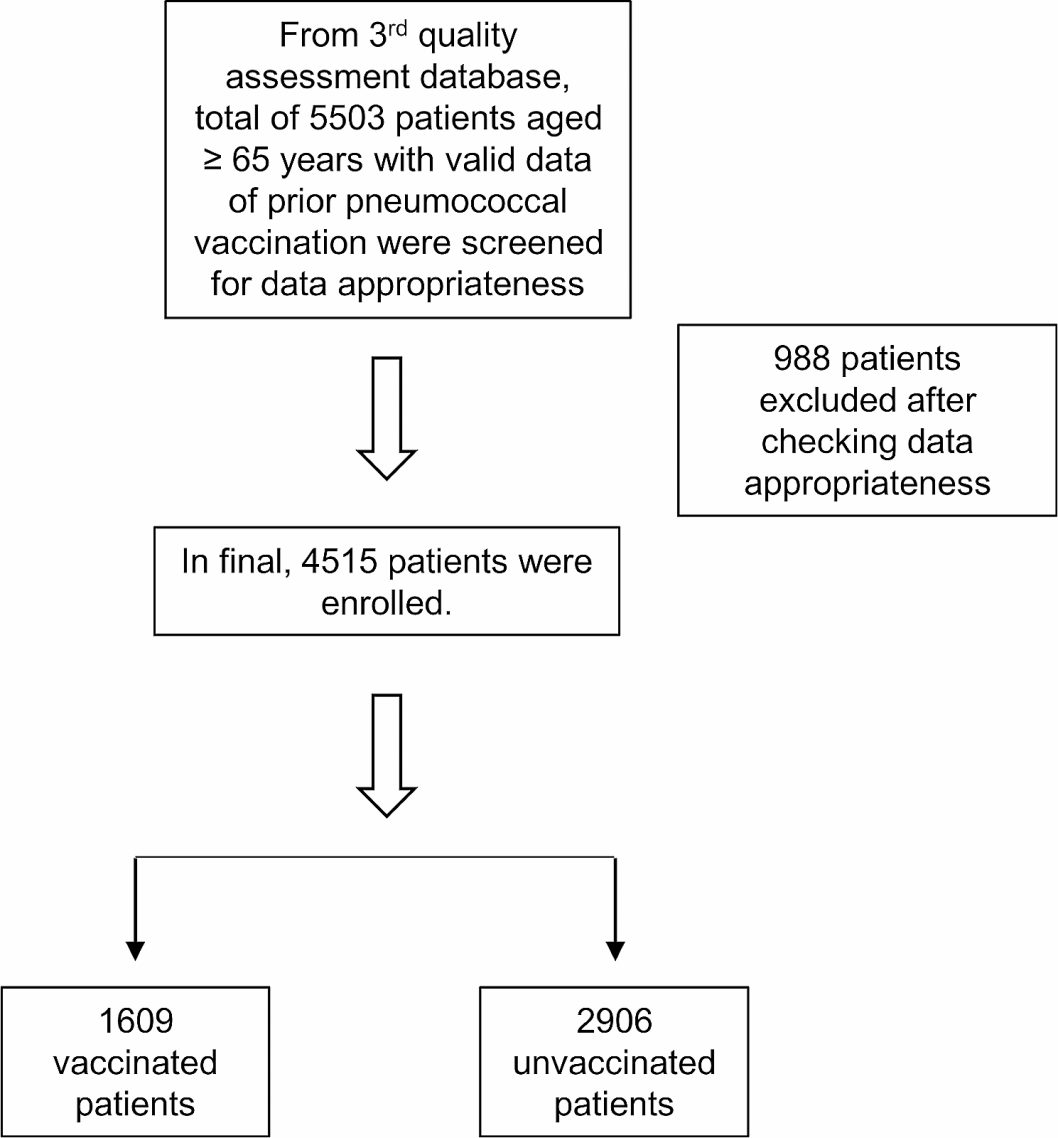
Flow diagram of study patients

A total of 5503 patients aged ≥ 65 years with valid data of prior pneumococcal vaccination were screened for the study, and 988 were excluded after checking the appropriateness of data. A total of 4515 patients were evaluated, of which 1609 patients were previously pneumococcal vaccinated and 2906 patients were unvaccinated (Fig. 1).

Among the total patients, mean age was 77.0 [71.0;82.0], and 2446 (54.2%) patients were male. The unvaccinated patients were older than pneumococcal vaccinated patients (Tables 1 and 78.0 vs. 76.0 years-old, *P* < 0.001). Mean CCI score was 3.0. Regarding type of admission, 4218 (93.4%) were admitted to general wards, while the other 6.6% were admitted to intensive care units (ICU). Mean hospital stay was 9.0 [7.0;13.0] days, and in-hospital mortality was 4.1%. 30-day mortality was 4.4%. Regarding comorbidities, chronic pulmonary disease was the most frequent (72.8%), followed by diabetes (39.5%), and mild liver disease (31.7%). Mean antibiotics treatment duration was 8 days. Regarding antibiotic regimens, 54.5% of the patients received beta-lactam only, followed by beta-lactam + quinolone combination (23.0%), quinolone only (13.3%) and beta-lactam + macrolide combination (9.1%).

**Table 1:** Comparison between pneumococcal vaccinated and unvaccinated elderly pneumonia patients

	Total (*N* = 4515)	Pneumococcal vaccinated (*N* = 1609)	Unvaccinated (*N* = 2906)	P-value
Age (years), mean (1^st^ quartile;3^rd^ quartile)	77.0 [71.0;82.0]	76.0 [71.0;82.0]	78.0 [72.0;83.0]	< 0.001***
Sex				
Male, n (%)	2446 (54.2)	907 (56.4)	1539 (53.0)	0.030*
Smoking status (*n* = 4340), n (%)				
Current	333 (7.7)	104 (6.8)	229 (8.2)	< 0.001***
Ex-smoker less than 1Y	65 (1.5)	28 (1.8)	37 (1.3)	
Ex-smoker more than 1Y	688 (15.9)	287 (18.7)	401 (14.3)	
Never	3254 (75.0)	1112 (72.6)	2142 (76.3)	
CURB-65				
Confusion (*n* = 4458), n (%)	238 (5.3)	61 (3.9)	177 (6.2)	0.001***
Urea (mg/dl) (*n* = 4515), mean [1^st^ quartile;3^rd^ quartile]	17.0 [13.0;22.5]	16.5 [13.0;21.8]	17.0 [13.0;23.0]	0.018*
Urea (>20 mg/dl), n (%)	1458 (32.3)	480 (29.8)	978 (33.7)	0.009**
SBP (mmHg) (*n* = 4458), mean [1^st^ quartile;3^rd^ quartile]	124.0 [110.0;140.0]	125.0 [110.0;140.0]	123.0 [110.0;140.0]	0.627
DBP (mmHg) (*n* = 4458), mean [1^st^ quartile;3^rd^ quartile]	71.5 [66.0;80.0]	72.0 [67.0;80.0]	71.0 [66.0;80.0]	0.957
SBP<90mmHg or /DBP≤60mmHg, n (%)	347 (7.7)	109 (6.8)	238 (8.2)	0.099
RR (rate/min), mean [1^st^ quartile;3^rd^ quartile]	20.0 [20.0;20.0]	20.0 [20.0;20.0]	20.0 [20.0;20.0]	0.002**
RR ≥30 rate/min, n (%)	77 (1.7)	20 (1.2)	57 (2.0)	0.096
CCI scores, mean [1^st^ quartile;3^rd^ quartile]	3.0 [2.0;5.0]	3.0 [2.0;5.0]	3.0 [2.0;5.0]	0.742
GW, n (%)	4218 (93.4)	1534 (95.3)	2684 (92.4)	< 0.001***
ICU, n (%)	297 (6.6)	75 (4.7)	222 (7.6)	
Hospital days, mean [1^st^ quartile;3^rd^ quartile]	9.0 [7.0;13.0]	9.0 [7.0;12.0]	9.0 [7.0;13.0]	0.038*
In-hospital improved, n (%)	4331 (95.9)	1571 (97.6)	2760 (95.0)	< 0.001***
30-day mortality, n (%)	197 (4.4)	42 (2.6)	155 (5.3)	< 0.001***
Comorbidities, n (%)				
MI	151 (3.6)	51 (3.4)	100 (3.8)	0.586
CHF	778 (18.6)	261 (17.3)	517 (19.4)	0.096
PVD	1100 (26.3)	393 (26.0)	707 (26.5)	0.732
Rheumatoid	236 (5.6)	85 (5.6)	151 (5.7)	1.000
Cerebrovascular disease	1061 (25.4)	374 (24.7)	687 (25.8)	0.479
Dementia	869 (20.8)	278 (18.4)	591 (22.2)	0.004**
Hemiplegia or paraplegia	74 (1.8)	22 (1.5)	52 (2.0)	0.295
Chronic pulmonary disease	3042 (72.8)	1117 (73.9)	1925 (72.2)	0.267
Chronic kidney disease	257 (6.2)	91 (6.0)	166 (6.2)	0.838
Mild liver disease	1325 (31.7)	486 (32.1)	839 (31.5)	0.684
Serious Liver disease	21 (0.5)	7 (0.5)	14 (0.5)	0.963
DM (with or without complication)	1785 (39.5)	653 (40.6)	1132 (39.0)	0.298
Any malignancy	380 (9.1)	140 (9.3)	240 (9.0)	0.827
Metastatic solid tumor	26 (0.6)	12 (0.8)	14 (0.5)	0.393
AIDS	0 (0.0)	0 (0.0)	0 (0.0)	-
Antibiotics				
Days, mean [1^st^ quartile;3^rd^ quartile]	8.0 [6.0;12.0]	8.0 [6.0;12.0]	8.0 [6.0;12.0]	0.102
Beta-lactam, n (%)	2462 (54.5)	889 (55.3)	1573 (54.1)	0.220
Quinolone, n (%)	600 (13.3)	204 (12.7)	396 (13.6)	
Beta-lactam + macrolide, n (%)	413 (9.1)	162 (10.1)	251 (8.6)	
Beta-lactam + quinolone, n (%)	1040 (23.0)	354 (22.0)	686 (23.6)	

*Abbreviations* SD = standard deviation, CURB-65 = confusion, uremia, respiratory rate, BP, age > 65 years, SBP = systolic blood pressure, DBP = diastolic blood pressure, RR = respiratory rate, CCI = Charlson comorbidity index, GW = general ward, ICU = intensive care unit, MI = myocardial infarction, CHF = congestive heart failure, PVD = peripheral vascular disease, DM = diabetes mellitus, AIDS = acquired immune deficiency syndrome, HIV = human immunodeficiency virus

Clinical parameters were compared between the vaccinated and unvaccinated groups. The unvaccinated group showed significantly higher mean age (78.0 vs. 76.0, *P* < 0.001), and had a higher proportion of current smokers (8.2 vs. 6.8%, *P* < 0.001). No statistically significant difference was observed in CCI scores. Regarding types of admission, a higher proportion of unvaccinated patients were admitted to ICU (7.6 vs. 4.7%, *P* < 0.001). The unvaccinated group showed significantly higher in-hospital mortality and 30-day mortality (5.0 vs. 2.4%, *P* < 0.001, and 5.3 vs. 2.6%, *P* < 0.001, respectively). When comorbidities were compared between the groups, the unvaccinated group showed a higher proportion of dementia (18.4 vs. 22.2%, *P* = 0.004), and no statistically significant differences were seen in other diseases. No significant differences were observed in duration of antibiotics treatment, or in antibiotics regimens (Table 1).

### Association with 30-day mortality and in-hospital mortality

**Fig. 2:**
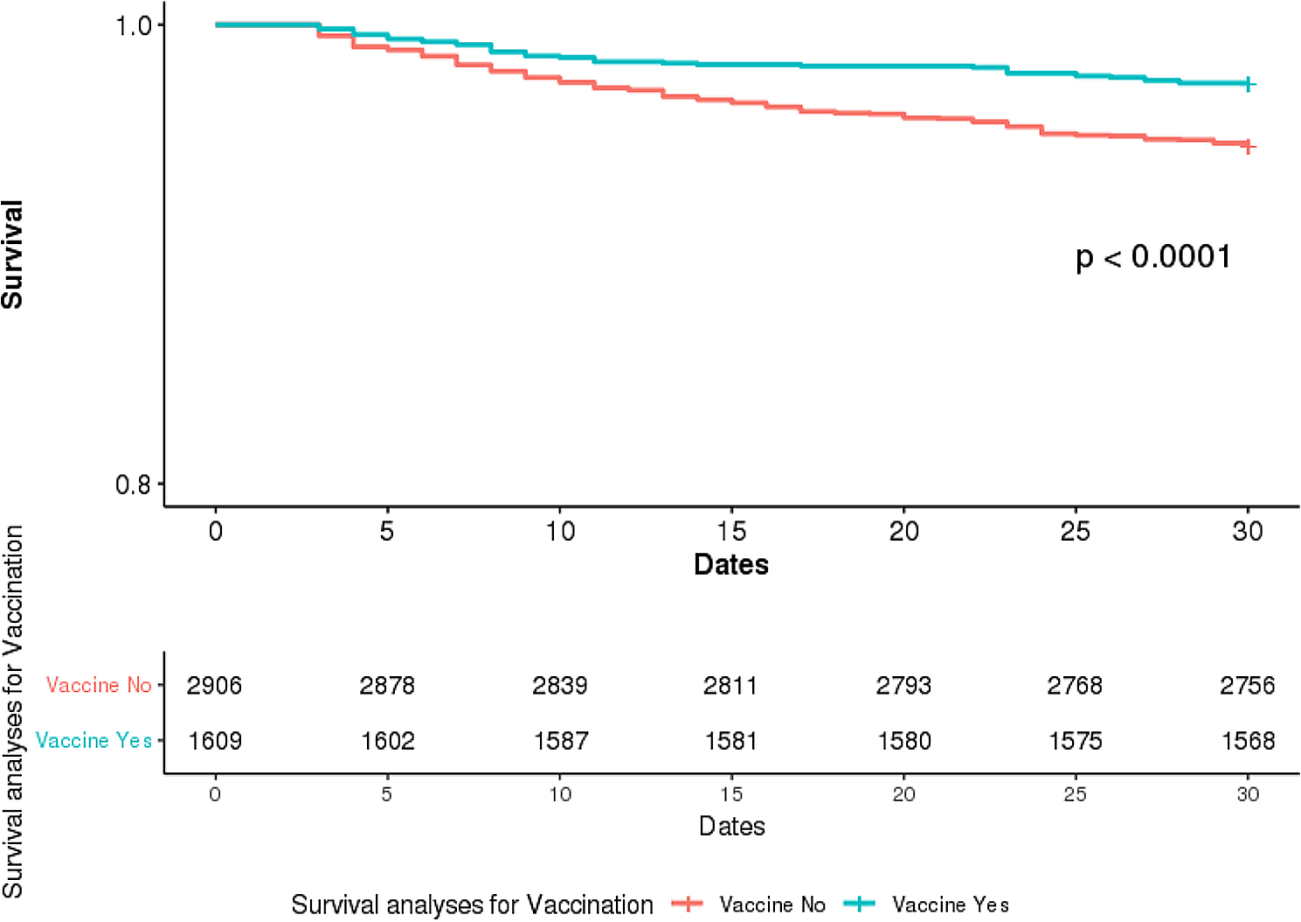
Comparison between pneumococcal vaccinated and unvaccinated of elderly pneumonia patient survival status within 30 days after hospital admission

We applied Cox proportional hazard model analysis for association with 30-day mortality. In the univariate analysis, age, gender, vaccination, CURB score and CCI were entered. Age (*P* < 0.001), vaccination (*P* < 0.001) and CURB score (*P* < 0.001 for both moderate and high) showed statistically significant association with in-30-day mortality. The vaccinated group showed significantly lower 30-day mortality when compared to the unvaccinated group (Fig. 2). In the multivariate analysis, in which all mentioned factors were entered, vaccination showed significant association with 30-day mortality (*P* = 0.002, adjusted hazard ratio [aHR] 0.576 (95% CI 0.409–0.812)). Age and moderate and high CURB score also showed significant association with 30-day mortality (Table 2).

**Table 2:** Univariate analysis and multivariate analysis on 30 days mortality (cox proportional hazard analysis)

	Univariate		Multivariate	
	Hazard ratio (95% confidence interval)	*P*-value	Hazard ratio (95% confidence interval)	*P*-value
Age	1.101 (1.081–1.121)	< 0.001***	1.076 (1.055–1.096)	< 0.001***
Female (vs. Male)	0.806 (0.607–1.071)	0.138	0.769 (0.576–1.027)	0.075
Prior vaccination, Yes (vs. No)	0.483 (2.071–0.343)	< 0.001***	0.576 (0.409–0.812)	0.002**
CURB score				
Low	Ref	Ref	Ref	Ref
Moderate	4.686 (3.215–6.831)	< 0.001***	3.561 (2.430–5.218)	< 0.001***
High	16.774 (11.227–25.061)	< 0.001***	11.507 (7.625–17.365)	< 0.001***
CCI score				
0	Ref	Ref	Ref	Ref
1	0.566 (0.312–1.028)	0.062	0.770 (0.423–1.403)	0.393
2+	0.802 (0.498–1.292)	0.365	0.995 (0.611–1.621)	0.984

CCI = Charlson comorbidity index, CURB = confusion, uremia, respiratory rate, blood pressure

We also performed logistic regression analysis for association with in-hospital mortality. Age, gender, vaccination, CURB score and CCI were entered. Age, vaccination and CURB score showed statistically significant association. In the multivariate analysis, in which all factors were entered, vaccination showed significant association with the in-hospital mortality (*P* = 0.001, odds ratio [OR] 0.534 (95% CI 0.367–0.778)). Age, male gender, and moderate and high CURB score also showed significant association (Table 3).

**Table 3:** Univariate analysis and multivariate analysis on in-hospital mortality (logistic regression analysis)

	Univariate		Multivariate	
	Odds ratio (95% confidence interval)	*P*-value	Odds ratio (95% confidence interval)	*P*-value
Age	1.102 (1.080–1.124)	< 0.001***	1.079 (0.056–1.103)	< 0.001***
Female (vs. Male)	0.751 (0.555–1.016)	0.064	0.708 (0.512–0.978)	0.036*
Prior vaccination, Yes (vs. No)	0.457 (0.318–0.657)	< 0.001***	0.534 (0.367–0.778)	0.001**
CURB score				
Low	Ref	Ref	Ref	Ref
Moderate	4.915 (3.287–7.349)	< 0.001***	3.748 (2.489–5.645)	< 0.001***
High	20.036 (12.942–31.018)	< 0.001***	14.260 (9.099–22.347)	< 0.001***
CCI score				
0	Ref	Ref	Ref	Ref
1	0.770 (0.404–1.467)	0.427	1.064 (0.531–2.132)	0.861
2+	0.940 (0.545–1.620)	0.824	1.108 (0.612–2.008)	0.735

CCI = Charlson comorbidity index, CURB = confusion, uremia, respiratory rate, blood pressure

### Association with hospital length of stay

Linear regression was performed to analyze the association with hospital LOS. In the simple analysis, age, gender, vaccination, CURB score, and CCI were entered. Age and CURB score were statistically significant factors, however, prior vaccination did not show statistical significance in the simple analysis (*P* = 0.075). In the multivariate linear regression analysis, in which all factors were entered, vaccination did not show statistical significance. Age and moderate and high CURB scores showed statistically significant association with hospital length of stay (Table 4).

**Table 4:** Simple and Multiple linear regression analyses on hospital length of stay

	Simple		Multiple	
	Exp (β) (95% confidence interval)	*P*-value	Exp (β) (95% confidence interval)	*P*-value
Age	1.103 (1.077–1.130)	< 0.001***	1.072 (1.046-1.100)	< 0.001***
Female (vs. Male)	0.864 (0.600-1.244)	0.432	0.931 (0.646–1.341)	0.700
Prior vaccination, Yes (vs. No)	0.708 (0.485–1.035)	0.075	0.829 (0.567–1.211)	0.332
CURB score				
Low	Ref	Ref	Ref	Ref
Moderate	5.438 (3.668–8.061)	< 0.001***	4.150 (2.771–6.214)	< 0.001***
High	24.840 (12.010-51.373)	< 0.001***	17.294 (8.288–36.087)	< 0.001***
CCI score				
0	Ref	Ref	Ref	Ref
1	1.211 (0.547–2.682)	0.637	1.361 (0.618–2.998)	0.445
2+	1.933 (0.963–3.880)	0.064	1.795 (0.898–3.588)	0.098

Exp (β) = exponential of beta, CCI = Charlson comorbidity index, CURB = confusion, uremia, respiratory rate, blood pressure

## Discussion

Using the cross-sectional nationwide data on the elderly population hospitalized due to CAP in South Korea, we showed that prior vaccination was associated with improved in-hospital mortality and 30-day mortality. There was a retrospective report in Japan that included 1355 patients which showed that pneumococcal vaccination reduced all-cause in-hospital mortality in elderly pneumonia patients [18], but according to our knowledge, few nationwide analyses have been reported. Despite the relatively short observation period (3 months), out study was the first investigation using nationwide cross-sectional data for admitted CAP patients.

It is recommended in South Korea that elderly people of 65 years or more receive pneumococcal vaccinations following the national vaccination program for the elderly population. There are two main kinds of pneumococcal vaccines available for the elderly: PCV13 and PPV23. In our study, prior vaccination showed beneficial effects in improving in-hospital and 30-day mortality in the elderly patients hospitalized due to CAP. It should be taken into consideration that our study includes all evaluable pneumonia cases regardless of pathogens (pneumococcal or non-pneumococcal). Nevertheless, prior pneumococcal vaccination was shown to improve 30 day-mortality with an HR of 0.576 and in-hospital mortality with an OR of 0.534. A retrospective observational study including 11 medical facilities in South Korea showed that streptococcus pneumoniae account for 7.4% of all caused CAP, and for 22.4% of pneumonia cases with identifiable pneumonia pathogens, which was reported in 2012 [3]. Other prospective cohort study in South Korea using data from 2015 to 2017 demonstrated that pneumococcal CAP comprised 9.4% of all caused CAP [19]. It is likely that in our study population, pneumonia pathogen distribution will be similar and that streptococcus pneumonia will account for 20–30% of the cases. It is possible that prior pneumococcal vaccination may have positive effects in the patients whose CAP was caused by pneumococci. We can make some assumptions as to how prior vaccination affected prognosis of the patients. An earlier study has shown that prior pneumococcal vaccination was associated with a more rapid resolution of pneumonia symptoms, and a lower risk of bacteremia [20]. Among the previously vaccinated patients, it is likely that the pneumococcal vaccines were effective in preventing pneumonia from aggravating to life-threatening status. Furthermore, pneumococcal vaccination might also affect viruses that cause pneumonia. In a previous study of pneumonia hospitalization in Western Australian children, hospitalization rates due to virus-specific pneumonia, such as respiratory syncytial virus, influenza A, and parainfluenza virus also decreased after universal PCV vaccination [21]. Past studies have demonstrated that spread of bacterial infection is promoted by viral infection in the pathogenesis of pneumonia; the viral infection disrupts mucociliary barrier in bronchial epithelium providing the proper environment for bacterial growth. PCV vaccination has beneficial effects on this superimposed bacterial coinfection after viral pneumonia, which was demonstrated in a previous trial [22]. One meta-analysis also reported that PPV23 showed efficacy against both invasive pneumococcal disease (IPD) and pneumococcal pneumonia in the patients aged ≥ 60 years, equivalent to PCV13 [23].

Vaccinated patients showed trend of shorter hospital stays when compared to the unvaccinated patients, however, in the linear regression model, vaccination did not show significant association with length of hospital stay. The retrospective study in Japan showed that prior pneumococcal vaccination was associated with shorter hospital stay and less medical expenditure [18]. It is possible that higher in-hospital mortality (especially 30-day mortality) in the unvaccinated group may have contributed to shorter hospital stay. A future in-depth analysis is required for clarification.

A prospective study in South Korea published in 2018 showed that vaccination rate was 33.3% in the IPD patients aged 65 or more [24]. In Germany, it was reported that the rate of pneumococcal vaccination in people ≥ 60 years old with IPD was 26% [25]. Among the population aged ≥ 65 years in South Korea, the pneumococcal vaccine coverage rate was reported to be 57.3% [9]. A cross-sectional questionnaire study in Japan published in 2018 showed that vaccination rate was 53.2% [26]. Considering that all our patients were hospitalized due to CAP, we can assume that if the population has more severe disease, it is more likely that the pneumococcal vaccination rate will be lower. It is uncertain how much preventive effect the prior pneumococcal vaccination has in the elderly population, and more validation studies are necessary.

Our study has some limitations. First, we could not acquire information on types of vaccines. No information was available on whether the vaccination was 13 or 23 valent, or if the vaccination was given before or after patients turned 65 years old. Second, we do not have data on patient’s influenza vaccination status. It is possible that patients who received pneumococcal vaccination also received influenza vaccination, and simultaneous preventions may have affected the mortality. Third, we could not collect the data about identification of causative microorganism. Fourth, propensity score matching necessary to reduce the confounding influence of baseline factors that exhibited significant differences between the vaccinated and unvaccinated groups were not conducted. Performing various statistical analysis were difficult due to the data being available for only a set duration of time. Factors such as the prevalence of dementia, which was more common in the unvaccinated group, may have impacted the clinical outcomes. Fifth, as more unvaccinated patients were admitted to ICU than vaccinated patients, this initial state might have affected the final outcome. Lastly, we could not adjust confounding factors of the study population. However, we conducted cox proportional hazard model and logistic regression analysis adjusting confounding factors between the two groups through multivariable analysis. It would be important to highlight that this study is worthy in respect of using real-world data.

## Conclusions

Prior pneumococcal vaccination in elderly populations is effective in improving in-hospital and 30-day mortalities among those hospitalized for pneumonia, however, it does not affect the length of admission duration.

## Data Availability

No datasets were generated or analysed during the current study.

## Abbreviations

CAP: community-acquired pneumonia
PCV13: 13-valent polysaccharide conjugate vaccine
PPV23: 23-valent pneumococcal polysaccharide vaccine
IPD: invasive pneumococcal diseases
QA: quality assessment
HIRA: Health and Insurance Review and Assessment Service
CCI: Charlson Comorbidity Index
LOS: length of stay
